# Innate immune memory in chronic HIV and HIV-associated neurocognitive disorders (HAND): potential mechanisms and clinical implications

**DOI:** 10.1007/s13365-024-01239-2

**Published:** 2024-12-28

**Authors:** Zachary Capriotti, Zachary Klase

**Affiliations:** 1 Department of Pharmacology and Physiology, Drexel University College of Medicine, Philadelphia, PA 19102 USA; 2 Molecular and Cell Biology and Genetics Graduate Program, Drexel University College of Medicine, Philadelphia, PA USA; 3 Center for Neuroimmunology and CNS Therapeutics, Institute for Molecular Medicine and Infectious Disease, Drexel University College of Medicine, Philadelphia, PA USA; 4 Sidney Kimmel Cancer Center, Thomas Jefferson University, Philadelphia, PA 19102 USA

**Keywords:** HIV-associated neurocognitive disorders, Neuroinflammation, Innate immune memory, Microglia, Monocytes, Cytokines

## Abstract

Although antiretroviral therapy (ART) has dramatically improved the outlook of the HIV/AIDS pandemic, people living with HIV (PLWH) on suppressive therapy are still at higher risk for a range of comorbidities including cardiovascular disease (CVD) and HIV-associated neurocognitive disorders (HAND), among others. Chronic inflammation and immune activation are thought to be an underlying cause of these comorbidities. Many of the factors thought to drive chronic inflammation and immune activation in HIV overlap with factors known to induce trained immunity. Trained immunity is a form of innate immune memory that metabolically and epigenetically reprograms innate immune cells to mount enhanced inflammatory responses upon secondary encounter with unrelated inflammatory stimuli. While this phenotype has been characterized in a variety of disease states in animals and humans, very little is known about its potential contribution to chronic HIV pathogenesis. In this review, a broad overview of innate immune memory in the periphery and the central nervous system (CNS) is provided and the evidence for trained immunity in the context of HIV is considered. In PLWH on ART, this phenotype could contribute to the chronic inflammation and immune activation associated with HIV comorbidities and could complicate HIV cure strategies due to the potential persistence of the phenotype after eradication of the virus. Further research into this immune state in the context of HIV may open the door for new therapeutics aimed at treating HIV comorbidities like HAND.

## Introduction

The development and widespread use of antiretroviral therapy (ART) has dramatically altered the course of the HIV/AIDS pandemic, fundamentally improving the prognosis of HIV infection from a near-certain progression to AIDS and subsequent mortality to a manageable chronic disease. Although ART inhibits viral replication, effectively reducing viremia to undetectable levels, and allowing for recovery of CD4 + T cell counts, it does not cure HIV infection due to the existence of persistent viral reservoirs. Further, people living with HIV (PLWH) who are on suppressive ART are at higher risk for a range of diseases including certain cancers, kidney and liver diseases, cardiovascular disease (CVD), osteopenia, osteoporosis, and other conditions compared to the HIV-uninfected population (Gallant et al. 2017; Lerner et al. 2020). In addition to these comorbidities, nearly half of PLWH experience HIV-associated neurocognitive disorders (HAND) which range in severity between asymptomatic neurocognitive impairment (ANI), mild neurocognitive disorder (MND) and HIV-associated dementia (HAD) (Wang et al. 2020a, b). Before the ART era, the majority of PLWH affected by neurocognitive impairments were diagnosed with the more severe HAD, while in the ART era, the majority are diagnosed with the less severe ANI, and HAD has become quite rare, although the overall prevalence of HAND in general remains mostly unchanged (Heaton et al. 2011; Wang et al. 2020a, b). Despite this improved situation, the milder forms of HAND still worsen quality of life and progression to more severe forms of the disorder is possible, especially as PLWH age (Grant et al. 2014; Sacktor et al. 2016; Spooner et al. 2022).

There are multiple proposed mechanisms that likely overlap to drive HAND and other comorbidities associated with HIV in the ART era including persistent viral gene expression, microbial translocation, and the toxicity of ART itself among others (Hsu and Sereti 2016; Saylor et al. 2016). Chronic inflammation and immune activation are thought to be a central thread that links these mechanisms and the comorbidities associated with them as multiple studies have revealed associations between the presence of these phenomena and morbidity and mortality in PLWH on ART (Grund et al. 2016; Hart et al. 2018; Hunt et al. 2016; Knudsen et al. 2016; Kuller et al. 2008; Wada et al. 2016). While a large focus of HIV research has been to elucidate the molecular mechanisms behind the ability of HIV to cause chronic inflammation, much is still unknown, and no therapeutic interventions have been successful in preventing inflammation and/or the comorbidities associated with it (Lv et al. 2021). Recently, the innate immune system has been shown to exhibit memory of previous inflammatory stimuli, altering the strength of subsequent responses to secondary insults in a non-specific manner (Netea et al. 2015). Trained immunity is one such memory phenotype classified by the ability of innate immune cells to be functionally reprogrammed by inflammatory stimuli to carry out a heightened immune response upon encounter with a secondary unrelated stimulus (Netea et al. 2016). While this phenotype is thought to have evolved as a protective mechanism against recurring infections, it is likely maladaptive in conditions of chronic inflammation where it can become dysregulated, leading to tissue damage (Bekkering et al. 2021). Despite the growing body of research exploring trained immunity in the context of multiple infectious and non-communicable diseases, its role in the chronic inflammation, immune activation, and subsequent comorbidities seen in PLWH on ART is poorly understood. In this review, a broad overview of innate immune memory phenotypes will be provided focusing on their existence in both the periphery and the CNS, and the current evidence for the existence of innate immune memory in the context of HIV will be reviewed along with analysis of the potential mechanisms and clinical implications of these phenotypes in HIV comorbidities like HAND.

## Innate immune memory

The concept of immunological memory has traditionally been attributed to the adaptive immune system alone, specifically regarding long-lived memory T and B lymphocytes (Crotty and Ahmed 2004). During an acute infection, some T and B cells recognize a specific epitope on the invading pathogen, then proliferate to mount an effective immune response and a portion of these populations become long-lived memory cells that retain antigen specificity, allowing for a more rapid and effective immune response upon a second encounter with that same pathogen. In contrast, the innate immune system has classically been understood as a broadly non-specific, first line of defense with no capacity for memory, which utilizes various pattern recognition receptors (PRRs) to detect pathogen- and/or damage-associated molecular patterns (PAMPs and DAMPs, respectively) as opposed to specific antigens (Janeway and Medzhitov 2002; Turvey and Broide 2010). In recent decades, this classical understanding has been challenged as memory-like phenotypes have been observed in innate immune cells like monocytes and macrophages (Quintin et al. 2012), neutrophils (Moorlag et al. 2020a, b), natural killer (NK) cells (Sun et al. 2009), microglia (Heng et al. 2021) and even non-immune cell types like skin epithelial stem cells (Naik et al. 2017). These cells have been shown to retain memory of previous immunogenic stimuli which reprogram them to carry out an altered inflammatory response to a secondary insult weeks, months, or even a year after the initial stimulus (Divangahi et al. 2021; Netea et al. 2011, 2020). Innate immune memory can be broadly categorized into two phenotypes: trained immunity and tolerance, of which, trained immunity will garner more attention in this review as it is better understood and likely more relevant in the context of chronic HIV infection. Trained immunity describes a heightened inflammatory response upon secondary stimulation while tolerance describes a dampened response, both of which are typically characterized by either increased or decreased proinflammatory cytokine production, respectively, among other altered effector functions like chemokine production and phagocytosis. Just as the innate immune system recognizes pathogens non-specifically, its capacity for memory is also non-specific, so trained or tolerant responses can be mounted against an immunogen that is completely unrelated to the initial stimulus, distinguishing this from the highly antigen-specific nature of memory in the adaptive immune system. It is thought that innate immune memory represents a more ancient form of immunological memory as similar phenotypes are observed in plants and invertebrates, which lack an adaptive immune system (Milutinovic and Kurtz 2016; Reimer-Michalski and Conrath 2016). This likely evolved as a protective mechanism against recurring infection or injury, as a heightened response upon secondary insult can help clear infection faster and a dampened response during a hyperinflammatory state will prevent tissue damage (Gourbal et al. 2018). However, as will be discussed later in this review, innate immune memory in the form of trained immunity can be maladaptive, especially in the context of chronic infection.

Multiple studies describing non-specific protective effects of certain vaccines and other immunogenic stimuli against unrelated infections inspired the conceptualization of trained immunity as these phenomena did not align with the classic antigen-specific model of adaptive immune memory. For example, the live-attenuated *Mycobacterium tuberculosis* vaccine, Bacille Calmette–Guérin (BCG), which has been widely used in humans since the early 20th century, has long been thought to confer protective effects that cannot be explained by its protection against *M. tuberculosis* alone (Chen et al. 2023). Epidemiological studies have suggested that BCG vaccination is associated with better overall survival for children in regions that experience high childhood mortality, partially due to its potential protection against malaria (Garly et al. 2003; Roth et al. 2005). It was also suggested that BCG vaccination resulted in a 43% reduction in infectious disease mortality rate in low-birthweight neonates (Biering-Sorensen et al. 2017). This phenomenon is not unique to BCG as epidemiological studies have observed non-specific protective effects imparted by other vaccines like diphtheria-tetanus-pertussis (DTP) and measles-containing vaccines (MCV) (Benn et al. 2013; Higgins et al. 2016). These findings are now thought to be a result of innate immune memory, with these stimuli priming innate immune cells to carry out a trained, or enhanced immune response against secondary insults (Chen et al. 2023). Studies performed in mice in the 1970s and ‘80s support this explanation as BCG vaccination protected mice against multiple unrelated pathogens like Babesia and *Plasmodium berghei* (Clark et al. 1976; Murphy 1981). Around the same time, several other studies reported phenomena functionally similar to trained immunity in mice, with stimuli such as *Corynebacterium parvum* and the fungal cell wall component, β-glucan, providing protection against subsequent infection with *Bordatella pertussis* and *Staphylococcus* aureus, respectively, weeks after the initial stimulus (Adlam et al. 1972; Di Luzio and Williams 1978). Later studies suggested similar non-specific protective effects were achieved independent of the adaptive immune system. For example, administration of a low-virulence strain of *Candida albicans* (PCA-2) in mice conferred significant protection against lethal inoculums of unrelated pathogens 2 weeks later, and this effect was shown in follow-up studies using athymic mice and selective pharmacological inhibition of T and B cell function to be mediated by macrophages but not cytotoxic T or B cells (Bistoni et al. 1986, 1988). A later study suggested that PCA-2-induced protection was dependent on increased pro-inflammatory cytokine production, a hallmark of what is now known as trained immunity, and that enhanced cytokine production capacity persisted for 2 weeks after PCA-2 training (Vecchiarelli et al. 1989). In a more recent study, equally enhanced clearance of *S. pneumoniae* was observed in T and B cell deficient SCID mice and normal mice after pre-treatment with bacterial flagellin, suggesting the innate immune system drives this non-specific protection (Munoz et al. 2010). At the time, trained immunity was not a concept these authors were aiming to elucidate, but it is now recognized that these findings represent early evidence of this phenotype, especially considering studies demonstrating the effect was T and B cell-independent.

Since these early studies suggesting the existence of innate immune memory, a large and growing body of research has identified these phenotypes in innate immune cells from humans and animal models in various contexts ranging from infectious disease and vaccination to non-communicable diseases. Innate immune memory in humans has been demonstrated both in vitro and in vivo where in vitro, innate immune cells are normally isolated and incubated with a primary stimulus, cultured for days or weeks in the absence of that stimulus, then incubated with a secondary stimulus and measured for cytokine secretion or other effector functions to determine whether memory of the first stimulus influenced the magnitude of the secondary response. The primary stimulus can also be administered in vivo, in the case of a vaccine like BCG, or an ongoing infection could be assumed to be the primary stimulus, like in chronic HIV, and innate immune cells are then isolated and incubated with a secondary stimulus ex vivo and measured for the altered response compared to those from individuals that did not encounter the primary stimulus. For example, in one of the earliest demonstrations of the trained response in humans, peripheral blood mononuclear cells (PBMCs) from healthy individuals inoculated with the BCG vaccine in vivo exhibited a heightened innate immune response including increased production of TNFα and IL-1β upon secondary challenge with unrelated pathogens ex vivo 3 months after the initial vaccination (Kleinnijenhuis et al. 2012). A similar experiment was done in the context of HIV, explained in more detail later, where innate immune cells were isolated from chronic HIV patients and the response to ex vivo secondary stimuli was compared to those from uninfected individuals (van der Heijden et al. 2021). Other in vivo studies in humans have revealed trained immunity phenotypes in malaria (Schrum et al. 2018), atherosclerosis (Bekkering et al. 2016), hypercholesterolemia (Bekkering et al. 2019), and autoinflammatory disorders (Bekkering et al. 2018), and studies in animal models have suggested the involvement of trained immunity in several other conditions. Further, an attempt to determine the ability of various bacterial, viral, and fungal stimuli to induce innate immune memory in isolated human monocytes in vitro revealed that the dose of the initial stimulus can determine whether a trained or tolerant response will be carried out upon secondary stimulation (Ifrim et al. 2014). Multiple stimuli including LPS, the TLR2 agonist Pam3CSK4, bacterial flagellin, and the viral dsRNA mimetic poly (I:C) were shown to induce a tolerant response at high doses and a trained response at low doses. This phenomenon has been well documented in the case of endotoxin tolerance, a state described as ‘immunoparalysis’ that occurs after excessive exposure to LPS during sepsis, characterized by a significantly dampened inflammatory response (Lopez-Collazo and del Fresno 2013; West and Heagy 2002). Underpinning this phenotype is the fact that at high doses, LPS induces tolerance, while at low doses training is induced (Morris and Li 2012). While innate immune memory can be induced in a variety of contexts based on the initial stimulus, most of the evidence for these phenotypes was demonstrated in monocytes and macrophages which are very short-lived in circulation, calling into question how innate immune memory could be stored for months at a time. Remarkably, a study in mice revealed that training with BCG occurs at the level of hematopoietic stem cells (HSCs) and multipotent progenitors (MPPs) in the bone marrow, enhancing myelopoiesis and programming HSCs to produce trained macrophages that cleared *M. tuberculosis* infection significantly better than untrained macrophages (Kaufmann et al. 2018). Another study reported very similar findings in the context of β-glucan training, suggesting the previous results were not unique to BCG and further implicating this mechanism as a factor driving long-term memory in innate immune cells (Mitroulis et al. 2018). Soon after these discoveries, reprogramming of HSCs in the bone marrow was confirmed in humans vaccinated with BCG, leading to the circulation of epigenetically reprogrammed monocytes for at least 90 days after vaccination (Cirovic et al. 2020). While more work should be done to further characterize this mechanism, it appears that innate immune memory is not just programmed at the level of mature circulating myeloid cells, but in the progenitor cells of the bone marrow, adding one explanation for the long-term memory observed.

The molecular mechanisms that underlie innate immune memory are not completely understood, but an intertwined mechanism of metabolic and epigenetic reprogramming is the dominating theory (Fanucchi et al. 2021). In an early demonstration of trained immunity in humans, 3 months after BCG vaccination, monocytes from BCG-treated individuals displayed increased occupancy of the active transcription marker H3K4me3 at TNFα and IL-6 promoters, suggesting a potential epigenetic mechanism driving the enhanced inflammatory response observed after vaccination (Kleinnijenhuis et al. 2012). Another study reported an increased enrichment of H3K4me3 at promoters for TNFα, IL-6, and IL-18 after training with β-glucan in vitro and remarkably, administering a histone methyltransferase inhibitor, MTA, during β-glucan training completely abolished the trained phenotype, suggesting epigenetic alterations are required for training (Quintin et al. 2012). Even before this, one study demonstrated that LPS-induced endotoxin tolerance in macrophages was driven in part by altered chromatin accessibility programmed by epigenetic marks such as H3K4me3 and AcH4 at promoters for genes involved in the inflammatory response (Foster et al. 2007). Since then, multiple studies have suggested epigenetic remodeling as an important mechanism driving innate immune memory, implicating marks such as H3K4me3, H3k4me1, and H3K27ac in multiple contexts of training with various stimuli including β-glucan, BCG, and endogenous inflammatory lipids (Arts et al. 2018; Bekkering et al. 2014; Cheng et al. 2014; Novakovic et al. 2016), but exactly how these translated to long-term memory was still not fully understood since epigenetic marks are inherently reversible. One study found that some epigenetic marks known to be important in innate immune memory, like H3K27ac, were lost at enhancers after the initial training stimulus was removed but H3K4me1 was retained at these same enhancers (Saeed et al. 2014). In other studies, H3K4me1 has been shown to facilitate faster deposition of H3K27ac and other active transcription marks upon restimulation since it maintains chromatin in an open state at the enhancers it interacts with, suggesting a potential mechanism by which trained cells retain ‘placeholders’ at inflammatory genes that are more rapidly transcribed upon secondary stimulus (Ostuni et al. 2013). Interestingly, mice lacking the enzyme Set7, which is known to deposit H3K4me1 at enhancers, were unable to store memory of previous β-glucan exposure and thus did not mount a trained response upon secondary stimulation (Keating et al. 2020a, b). While this is one mechanism that could facilitate long-term epigenetic memory, it is still unclear how other epigenetic marks are maintained, considering observations that some like H3K4me3 are durable for at least 3 months after training (Kleinnijenhuis et al. 2012). From the evidence described above, it was clear that epigenetic modifications were involved in trained immunity, but how they are deposited at specific loci for inflammatory genes, as opposed to global deposition, is still not fully understood. There is some evidence that three-dimensional chromatin structures known as topologically associated domains (TADs) can organize innate immune genes into distinct regions which are then targeted by immune gene-priming long noncoding RNAs (IPLs) that direct epigenetic enzymes to these sites for modification (Fok et al. 2018). One IPL named upstream master lncRNA of the inflammatory chemokine locus (UMLILO) has been identified as a key player in directing the deposition of H3K4me3 at promoters of genes associated with trained immunity (Fanucchi et al. 2019). Interestingly, increased expression of UMLILO and IPL-IL1, which is known to regulate IL-1β expression, was observed in BCG-trained neutrophils, and donors displaying the highest level of trained immunity exhibited the highest IPL expression (Moorlag et al. 2020a, b). While understanding these mechanisms is a step in the right direction, the intricacies of epigenetic remodeling in innate immune memory are still being uncovered.

In addition to epigenetics, metabolic reprogramming is implicated in the induction of innate immune memory, and multiple studies have begun to uncover mechanistic links between these two pillars of trained and tolerant immune responses (Riksen and Netea 2021). For example, β-glucan trained monocytes display a shift from oxidative phosphorylation toward aerobic glycolysis, also known as the Warburg effect, marked by increased glucose consumption, high NAD+/NADH ratio, and increased lactate production, and this shift is mediated via an Akt-mTOR-HIF-1α pathway (Cheng et al. 2014). Inhibition of this pathway abolished β-glucan-induced trained immunity, pointing to the importance of metabolic reprogramming in establishing the phenotype. Aside from providing energy, it is thought that certain metabolites produced in aerobic glycolysis and other interconnected metabolic pathways can act as substrates or cofactors for downstream epigenetic remodeling driving innate immune memory (Fanucchi et al. 2021; Riksen and Netea 2021). For example, it has been shown that β-glucan trained monocytes experience an accumulation of the metabolic product, fumarate, which inhibits KDM5 histone demethylases (known to demethylate H3K4), suggesting a mechanism whereby this metabolite protects trained immunity-associated epigenetic markers from erasure by KDM5 (Arts et al. 2016). Regulation in the other direction also seems to occur, as β-glucan training is accompanied by upregulation of KDM4 demethylases, which are known to play a role in facilitating HIF-1 target gene transcription leading to downstream metabolic changes (Moorlag et al. 2022). It has also been observed that cholesterol synthesis is upregulated during trained immunity, and a metabolite from this pathway, mevalonate, was shown to be an important factor driving trained immunity in monocytes (Bekkering et al. 2018). Interestingly, stimulation with mevalonate itself was able to induce trained immunity and inhibition of the cholesterol synthesis pathway with statins blocked β-glucan-induced training. Although it is not completely clear how mevalonate facilitates epigenetic changes, a global epigenetic signature similar to what is seen in β-glucan training was observed when monocytes were trained with mevalonate alone, further suggesting a link between changes in metabolic pathways and epigenetic reprogramming. Additionally, the metabolite produced in glutaminolysis and the citric acid cycle, α-ketoglutarate, has been shown to interact with the histone demethylase JMJD3 to facilitate epigenetic reprogramming in normal macrophage activation and α-ketoglutarate has also been implicated as an important factor driving endotoxin tolerance, albeit not through JMJD3 (Liu et al. 2017). While the full range of mechanisms driving innate immune memory is still being uncovered, metabolic and epigenetic reprogramming are essential for inducing innate immune memory phenotypes, and there is evidence that these mechanisms are intimately intertwined.

## Innate immune memory in the central nervous system

Compared to the recent advances in characterizing innate immune memory phenotypes in the periphery, much less is known about this phenomenon in the CNS. The CNS is commonly referred to as “immune privileged,” which often evokes misconceptions that it is immunologically passive and completely isolated from immune responses in the periphery (Carson et al. 2006). While some aspects of this classic description remain true, it is now understood that there is much more active immune surveillance occurring in the CNS than previously thought, especially from microglia, the yolk-sac derived CNS-resident myeloid cells that represent the major innate immune cell type in the brain (Kettenmann et al. 2011). Microglia display a ramified morphology in their resting state with processes that extend from a cell body, distinguishing them from other tissue-resident macrophages. In this ‘resting’ state, microglia utilize their processes to actively survey their microenvironment, in direct contrast with their original characterization as a mere component of the ‘glue’ between neurons (Allen and Barres 2009; Nimmerjahn et al. 2005). Upon encounter with a PAMP, DAMP, or other signals indicating potential danger to the CNS, microglia can enter an ‘activated’ state, displaying significantly altered gene expression, amoeboid morphology, proinflammatory cytokine production, recruiting of other immune cells, and utilization of phagocytosis among other effector functions to help clear an infection or resolve an injury (Kettenmann et al. 2011; Kierdorf and Prinz 2013). While the dynamics of microglia activation have been studied for decades, investigations into the potential for a memory-like phenotype in these cells are relatively new. Exploration of innate immune memory in the CNS began with the characterization of ‘microglia priming’ which is a phenotype functionally similar to trained immunity in the periphery, but which was actually conceptualized independently (Neher and Cunningham 2019). Initial investigations which eventually led to the discovery of microglia priming were focused on mechanisms of exaggerated neuroinflammation in the context of aging as well as neurodegenerative diseases like Alzheimer’s and Parkinsons with the idea that microglia were being ‘primed’ by the neurodegenerative state of the brain in these conditions (Cunningham et al. 2005; Godbout et al. 2005; Perry et al. 2007; Perry and Holmes 2014). Like trained immunity in the periphery, ‘primed’ microglia have been described as hyperresponsive to secondary stimuli after being trained by a prior initial stimulus, although much less is known regarding the molecular mechanisms driving this functional phenotype (Haley et al. 2019). As in cells of the periphery, microglia have also been shown to display a ‘tolerant’ response upon secondary stimulation after initial exposure to LPS (Brown 2019). Since microglia priming and innate immune memory phenotypes of the periphery are functionally similar, Neher and Cunningham have proposed an integration of their nomenclature which will be used throughout this review; thus microglia are ‘primed’ by an initial stimulus to carry out a ‘trained’ response, or are ‘desensitized’ by an initial stimulus to carry out a ‘tolerant’ response (Neher and Cunningham 2019).

The early research that led to the characterization of innate immune memory in the CNS was not necessarily focused on a memory-like phenotype programmed from a past challenge, as it was in the periphery, but focused more on the superimposition of secondary challenges onto ongoing chronic neurodegenerative and inflammatory states. Evidence that peripheral infection can stimulate synthesis of proinflammatory cytokines in the CNS (Laye et al. 1994; Perry et al. 2007; Pitossi et al. 1997), along with observations that Alzheimer’s patients experience worse behavioral complications upon peripheral infection compared to healthy elderly individuals, led to the idea that peripheral infection during ongoing chronic neurodegeneration causes an exaggerated proinflammatory response in the CNS, explaining the exacerbated behavioral outcomes seen in Alzheimer’s patients (Combrinck et al. 2002). The ME7 murine model of prion disease was utilized to recapitulate the chronic neurodegeneration and microglial activation seen in Alzheimer’s disease and in this state, exaggerated sickness behavior was observed upon peripheral stimulation with LPS and these behavioral changes were accompanied by an exaggerated IL-1β response in the brain (Combrinck et al. 2002). These results served as the basis for the original hypothesis of microglia priming, as it was thought that the observed hyperinflammatory response was driven by microglia that had been “primed” by ongoing neurodegeneration. This hypothesis was tested in the same murine model where either central or peripheral LPS challenges were superimposed onto prion-diseased mice in vivo (Cunningham et al. 2005). In contrast to the initial study which only examined the IL-1β response (Combrinck et al. 2002), systemic LPS challenge induced increased transcription of a wider array of proinflammatory mediators including IL-1β, TNFα, IL-6, and inducible nitric oxide synthase (iNOS) in brains of prion-diseased mice compared to healthy controls (Cunningham et al. 2005). However, the role of microglia in this response was not fully confirmed as gene transcription was measured from whole brain homogenates as opposed to a purified microglia population, hindering the ability to rule out a contribution from infiltrating immune cells or other macrophage populations in the brain. The study did however suggest the role of microglia in the exaggerated proinflammatory response based on immunohistochemical staining of brain slices which showed increased IL-1β staining localized to activated microglial cells in LPS-stimulated, prion-diseased mice. Around the same time, a similar line of investigation was carried out to elucidate the mechanisms behind delirium (acute cognitive impairments) in elderly patients during peripheral infections. Normal aging mice were utilized to recapitulate the elderly human brain and proinflammatory responses were measured after in vivo LPS stimulation both peripherally and centrally (Godbout et al. 2005; Huang et al. 2008). Aged mice exhibited an exaggerated response to peripheral LPS challenge characterized by increased proinflammatory cytokine production and expression of proinflammatory genes, supporting the idea that aging leads to the priming of microglia for a trained response. Although these changes were measured from whole brain homogenates, and thus did not conclusively implicate microglia as the cell type driving the observed response, a follow-up study by the same group more thoroughly demonstrated the effect was microglia-dependent, confirming increased proinflammatory gene expression and cytokine production in isolated microglia populations from aged mice stimulated with LPS in vivo (Henry et al. 2009). While these pioneering works that helped develop the concept of microglia priming were focused on aging and chronic neurodegenerative diseases like Alzheimer’s, similar phenotypes have been reported in models of Parkinson’s disease (Pott Godoy et al. 2008), Wallerian axon degeneration (Palin et al. 2008), multiple sclerosis (Ramaglia et al. 2012), early life infection (Bilbo 2010), systemic infection *in utero* (Cao et al. 2015), stress (Frank et al. 2007), and systemic fungal infections (Heng et al. 2021) suggesting these conditions themselves, or the inflammatory and/or neurodegenerative states caused by these conditions, could prime microglia for a trained immune response.

Since most of the previously mentioned studies focused on the altered response of microglia after secondary insult during ongoing chronic disease or aging, the capacity for memory in these cells was not necessarily explored. The question remained whether microglia would respond in a similar fashion due to a stored memory of previous insult after returning to a steady state, like what has been shown for innate immune cells of the periphery. In a landmark study, evidence for a memory-like response in microglia was demonstrated in mice after varying doses of LPS (Wendeln et al. 2018). Two different programs of peripheral LPS injection were shown to induce either training or tolerance in the CNS determined by microglia proinflammatory cytokine production. Proinflammatory cytokines were measured from whole brain homogenate, but microglia-specific knockout of Tak1, a regulator of NF-κB, JNK, and ERK1/2 pathways (Goldmann et al. 2013), or histone deacetylases 1 and 2, regulators of epigenetic reprogramming for macrophage inflammatory responses (Datta et al. 2018), almost completely blocked the previously observed memory responses, pointing to microglia as the drivers of these phenotypes (Wendeln et al. 2018). Interestingly, the authors detected no LPS in the brain parenchyma nor did they detect any evidence of blood-brain barrier (BBB) disruption or extravasating peripheral myeloid cells, suggesting the observed responses were a result of some signaling from the periphery to the brain rather than a direct contact of microglia with the LPS itself, and infiltrating myeloid cells were not the drivers of the observed memory response. Furthermore, the authors demonstrated that the tolerant response was associated with alleviated neuropathology, while the trained response was associated with worsened neuropathology in the context of stroke as well as the APP23 murine model of Alzheimer’s disease. APP23 mice that had been administered the trained immunity-inducing LPS program 3 months prior to onset of amyloid-β (Aβ) plaque deposition exhibited significantly increased plaque load and total Aβ levels as well as increased production of IL-1β, IL-6, and IL-12 in the brain. Similar outcomes were observed after stroke was induced in mice treated with the trained immunity-inducing LPS program 1 month prior as these mice exhibited more proinflammatory cytokine production, more microglial activation, and subsequent neuronal damage. Intriguingly, epigenetic markers of active transcription were observed at enhancers for HIF-1 signaling pathway genes in trained microglia (Wendeln et al. 2018), suggesting a similar epigenetic and metabolic mechanism to what has been described in the periphery (Cheng et al. 2014). Furthermore, the upregulation of HIF-1 signaling in trained microglia promoted a shift toward glycolysis as measured by increased mitochondrial membrane potential and lactate production, a function known to represent the metabolic basis for trained immunity in the periphery (Cheng et al. 2014). From this evidence, it has become clearer that microglia have the capacity to be primed or desensitized by various stimuli to carry out trained or tolerant responses, and these can have protective or maladaptive effects in the context of certain neurodegenerative states, aligning this phenotype with what has been described in the periphery.

While the previously described evidence suggests a shared epigenetic and metabolic mechanism of innate immune memory between microglia and cells of the periphery (Wendeln et al. 2018), several studies have further implicated metabolic and epigenetic rewiring as well as other mechanisms that may act in parallel. For example, in the context of normal aging, downregulation of CX3CR1 was proposed as a potential mechanism for the trained microglia response after peripheral LPS stimulation (Wynne et al. 2010). CX3CR1 is constitutively expressed on the surface of microglia in the healthy brain and interacts with CX3CL1 expressed by neurons serving as a constant inhibitory signaling interaction that promotes a “resting” homeostatic microglial phenotype (Cardona et al. 2006; Mattison et al. 2013). Thus, the downregulation of CX3CR1 in the aged brain could lead to a general bias toward the active state and while this does not point directly to a mechanism of metabolic and epigenetic rewiring, this could act in parallel (Wynne et al. 2010). Indeed, downregulation of CXC3R1 was shown to occur in trained microglia in other disease contexts in a gene expression study meta-analysis discussed in further detail later (Holtman et al. 2015). Other studies provide more evidence for the epigenetic mechanism of innate immune memory in microglia. For example, microglia isolated from aged mice displayed hypomethylation of DNA at the IL-1β promoter which was associated with increased IL-1β production and prolonged sickness behavior after peripheral stimulation with LPS (Matt et al. 2016). In a different study, the microglia tolerance response was driven by reduced active epigenetic markers like AcH3 and H3K4me3 at the promoters of IL-1β and TNFα as well as increased repressive epigenetic marker H3K9me2 at the IL-1β promoter (Schaafsma et al. 2015). In a murine model of microglia training via accelerated aging, the active epigenetic markers H3K27ac, H3K4me3, and H3K4me1 were enriched at many genes upregulated after priming including genes involved in “inflammatory response”, “regulation of cytokine production”, and “innate immune response” pathways and the repressive histone marker, H3K27me3 was depleted in many of these promoters (Zhang et al. 2022). H3K27ac deposition was also implicated as a potential mechanistic factor in microglial training in a murine model of Parkinson’s disease (Huang et al. 2023). Other studies have shed more light on the potential metabolic mechanisms of innate immune memory in microglia. For example, in a murine model of Alzheimer’s disease, microglia tolerance was accompanied by defective glycolytic metabolism and a decrease in signaling along the AKT-mTOR-HIF-1α pathway (Baik et al. 2019), similar to what has been shown in myeloid cells of the periphery as well as microglia (Bekkering et al. 2018; Cheng et al. 2014; Wendeln et al. 2018). Further, PI3Kγ/AKT signaling was shown to be important for neonatal microglia training through LPS priming (Lajqi et al. 2019), similar to what has been shown in trained immunity in the periphery (Cheng et al. 2014). While epigenetic and metabolic mechanisms for innate immune memory in microglia have been suggested by these studies, questions remain regarding how long memories of inflammatory insult can be stored by microglia, and whether the mechanisms already suggested would mediate that long-term memory.

Like innate immune memory in the periphery, microglial training and tolerance are largely demonstrated with functional assays displaying altered cytokine production among other effector functions. This characterization requires live microglia that can be stimulated and restimulated in vitro, or preclinical animal models which can be stimulated in vivo to then analyze cells from whole harvested brain. This severely limits the scope of these studies as this method of characterizing microglia cannot be easily carried out in humans for obvious ethical and practical reasons, which is the reason innate immune memory in microglia has not been well documented in humans while in the periphery, it has. On top of this, trained or tolerant microglia cannot be easily identified from postmortem CNS samples as there is no widely accepted gene expression signature characteristic of these phenotypes, hindering the ability to correlate the presence of trained or tolerant microglia with disease outcomes in humans. Considering this, several attempts have been made to elucidate a gene expression signature characteristic of trained and tolerant microglia. For example, a meta-analysis of multiple transcriptomics studies that examined trained microglia phenotypes in various mouse models including aging, Alzheimer’s disease, and amyotrophic lateral sclerosis (ALS) utilized weighted gene co-expression network analysis comparing the expression signature of trained microglia to a microglia phenotype of acute activation (Holtman et al. 2015). Using this analysis, several genes were identified as commonly expressed in trained, but not acutely activated microglia across the various models including Itgax (CD11c), Lgals3, Axl, Clec7a (Dectin-1), Cxcr4, and notably, MHC II. Interestingly, Neher and Cunningham point out that some of these putative trained microglia markers proposed by Holtman et al. overlap with markers characteristic of the damage associated microglia (DAM) phenotype that was shown to be important in Alzheimer’s disease pathogenesis, and because of this, some speculate that the DAM phenotype could be representative of trained microglia that were primed by the neurodegenerative conditions in the brain during Alzheimer’s progression (Keren-Shaul et al. 2017; Neher and Cunningham 2019). An earlier study also proposed increased MHC II expression as a marker for trained microglia in the context of aging (Godbout et al. 2005). In aged microglia, which have been shown to exhibit a trained phenotype, MHC II is upregulated (Godbout et al. 2005; Norden and Godbout 2013; Rogers et al. 1988). MHC II is also upregulated in normal microglia activation (Jurga et al. 2020), but this is accompanied by increased expression of proinflammatory cytokine genes like IL-1β and IL-6 (Godbout et al. 2005). Thus, in normal microglia, MHC II and proinflammatory cytokine genes are downregulated in the resting state while upregulation of both MHC II and proinflammatory cytokines occurs during activation. However, in aged microglia, MHC II is upregulated in the resting state while IL-1β and IL-6 are maintained at low levels. This means that a signature of MHC II^high^/IL-1β^low^/IL-6^low^ could indicate trained microglia. Aside from these proposed markers, a thoroughly validated and widely accepted signature is yet to be established that is standard across models.

## Evidence for innate immune memory in the context of HIV

**Fig. 1:**
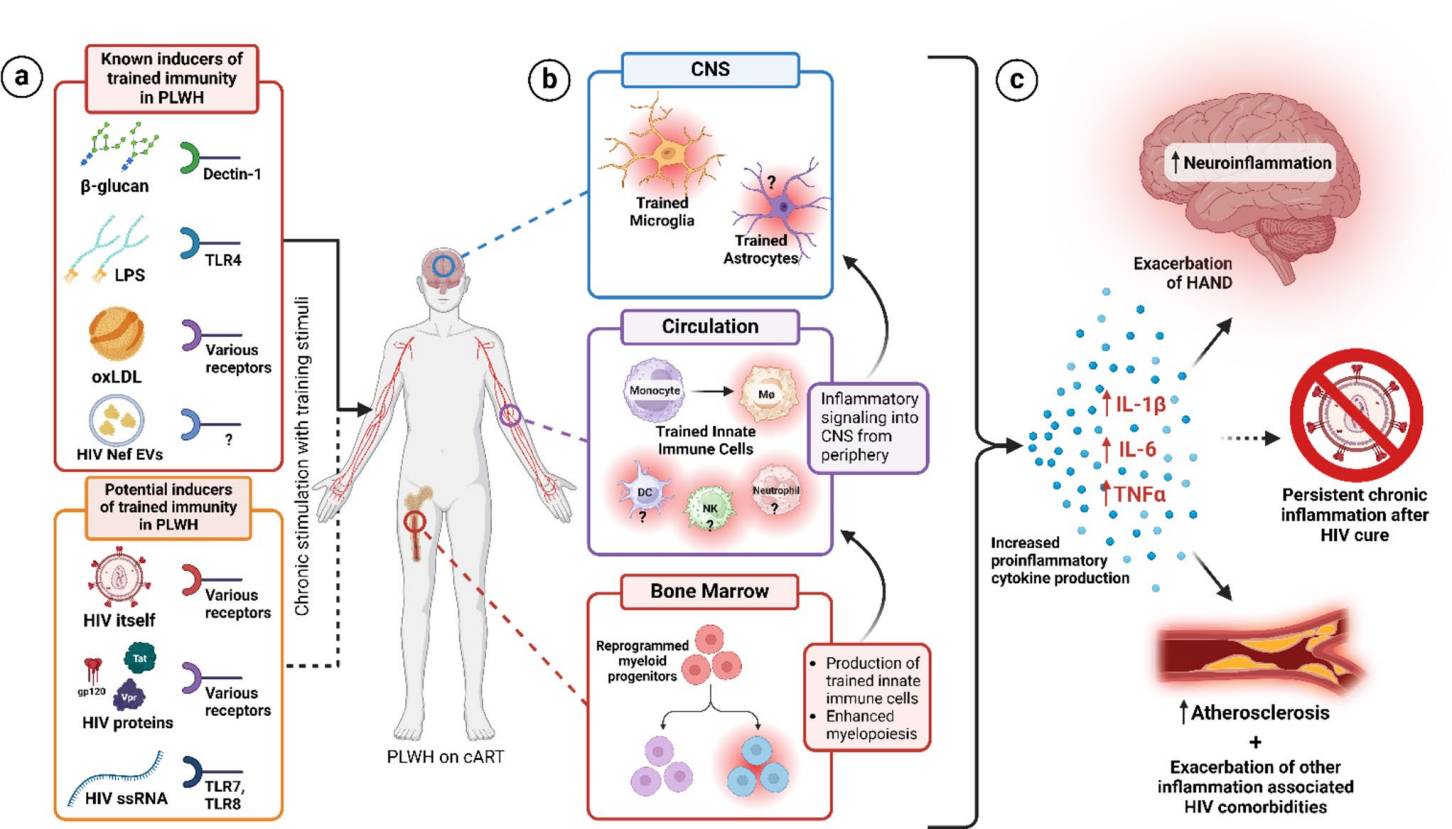
Proposed model for the induction of trained immunity in PLWH on ART and potential clinical implications of this phenomenon. **a**) known inducers of trained immunity that have been shown to be circulating at levels higher than normal in PLWH on ART as well as factors in PLWH on ART that have not yet been confirmed as trained immunity inducers, but which studies have shown to cause phenotypes analogous to trained immunity. **b**) the compartments and cell types that would be affected by these known and potential inducers of trained immunity circulating in PLWH on ART and how interactions between these compartments could perpetuate trained immunity phenotypes. **c**) the putative outcomes of persistent trained immunity phenotypes and their potential clinical implications

Although it has recently been proposed that trained immunity could be involved in HIV pathogenesis in the ART era (Sviridov and Bukrinsky 2023; Sviridov et al. 2022), very few studies have explicitly investigated this possibility. In one of the first studies to explore trained immunity in HIV, ex vivo stimulation of monocytes from PLWH on ART with various bacterial, fungal, and viral stimuli resulted in increased production of proinflammatory cytokines IL-1β, TNFα, and IL-6 compared to monocytes from uninfected healthy controls, suggesting the presence of a trained immunity phenotype (van der Heijden et al. 2021). These enhanced proinflammatory cytokine responses were associated with increased biomarkers of persistent inflammation in serum from these patients, suggesting the state of chronic inflammation seen in PLWH on ART could be a cause of, or be caused by, trained immunity. Remarkably, longitudinal sampling of the original cohort revealed that the enhanced monocyte response observed was not transient, as equivalent responses were measured in the same blood donors 1 year later. The study also suggested circulating β-glucan, which is known to be detectable in serum of PLWH on ART (Mehraj et al. 2020), as a potential stimulus driving the trained phenotype, as increased ex vivo IL-1β responses and IL1B gene expression were correlated with higher serum β-glucan levels (van der Heijden et al. 2021). In a follow-up study exploring the mechanisms of this observed phenotype, gene expression signatures of trained monocytes from a small subset of the same cohort of PLWH revealed upregulation of general innate and myeloid proinflammatory gene programs partially characterized by upregulation of IFN-mediated pathways (Knoll et al. 2023). However, since a common gene expression signature characteristic of trained immunity in monocytes does not exist, this study was not able to link the unique gene expression signature of monocytes from PLWH to that of trained monocytes. In this same study, ATAC-seq analysis of monocytes from PLWH did not reveal any differentially accessible regions compared to uninfected controls, as would be expected in trained monocytes (Saeed et al. 2014; You et al. 2021), although the very small sample size in this study likely prevented the identification of significant differences. In light of this, the authors plan to apply ATAC-seq and other powerful bulk and single-cell ‘omics techniques to a much larger cohort of PLWH to better understand whether epigenetic reprogramming plays a role in the trained phenotype they observed, allowing for a deeper investigation into the molecular mechanisms of HIV-induced trained immunity (Vos et al. 2022). Aside from this, another study which explicitly examined trained immunity in the context of HIV demonstrated that extracellular vesicles (EVs) carrying HIV Nef, which are known to be secreted from HIV-infected cells (McNamara et al. 2018), induced trained immunity in macrophages (Dubrovsky et al. 2022). Specifically, human primary monocytes from uninfected donors were exposed to EVs carrying HIV Nef, then after a 6-day rest/macrophage differentiation period, a secondary stimulus of LPS induced increased production of TNFα and IL-6 compared to cells that were not exposed to HIV Nef. This phenotype was also demonstrated in vivo where mice were injected with Nef EVs intravenously and their response to secondary stimulation ex vivo was shown to display a trained phenotype. The trained phenotype was also shown to be quite durable in vivo as bone marrow from Nef EV-trained mice was transplanted into naïve mice and a trained response to ex vivo LPS stimulation was seen 11 weeks after the transplant in naïve mice. Interestingly, increased cholesterol biosynthesis and IGF1R signaling were observed in these trained macrophages, similar to what has been shown in the context of β-glucan training where IGF1R signaling activates mTOR leading to increased glycolysis which then increases the production of substrates for epigenetic modifications, implicating metabolic reprogramming as a common mechanism contributing to training in these different contexts (Bekkering et al. 2018). These studies together suggest that trained immunity could be induced in PLWH on ART, providing two likely mechanisms for the training stimuli including circulating β-glucan and secreted EVs carrying HIV Nef (Fig. 1a). In addition to these studies which examined potential inducers of trained immunity in the context of HIV, another study demonstrated that in addition to the trained immunity phenotype observed in monocytes and macrophages from PLWH, macrophages from PLWH also display an impaired ability to establish endotoxin tolerance in response to LPS (Faia et al. 2021). This phenomenon was shown to be driven by decreased expression of the transcription factor IKZF1 (IKAROS), which is thought to be involved in the induction of anti-inflammatory signaling in response to high doses of LPS (Sung et al. 2014). Interestingly, a follow-up study from the same group revealed that MDMs from PLWH on ART display metabolic reprogramming partially defined by increased utilization of glycolysis (Vittori et al. 2024), similar to what has been shown in innate immune cells trained by other stimuli like β-glucan and BCG (Arts et al. 2016; Cheng et al. 2014). These findings were also in agreement with another study which showed that HIV infection is associated with increased utilization of glycolysis and the pentose phosphate pathway (PPP), as well as increased production of lactate (Deme et al. 2022), which have all been shown to be associated with trained immunity in other contexts (Riksen and Netea 2021).

Before these studies which explicitly examined trained immunity in the context of HIV, some studies in SIV/HIV vaccine candidates revealed a training-like state caused by immunization, suggesting that HIV itself or components of HIV could potentially induce trained immunity. Upon immunization with an SIV DNA vaccine, enhanced protection against subsequent SIV infection was correlated with the vaccine’s ability to induce upregulation of the IL-1β pathway in monocytes, and the authors suggested this could represent an induction of trained immunity (Vaccari et al. 2018). In a similar study, mucosal administration of HIV/SIV peptide vaccines in macaques did not elicit a humoral response against the HIV envelope glycoprotein, gp120, and the gag- and env-specific T cell responses that were measured did not correlate with the animals’ protection against SHIV infection, suggesting an adaptive immune system mediated response was not conferring protection (Sui et al. 2019). Instead, a myeloid cell response was measured that did correlate with protection against SHIV infection, which led the authors to investigate whether a trained immunity phenotype was at play. They found that ex vivo stimulation of myeloid cells from vaccinated animals resulted in increased production of TNFα and IL-6, and this response was correlated with protection against SHIV infection in vivo, leading them to suggest that trained immunity may have conferred enhanced protection in the vaccinated animals. Despite these interesting findings suggesting trained immunity could enhance HIV vaccine protection, another study utilizing an attenuated BCG vaccine expressing SIV antigens had the opposite effect (Derrick 2017; Jensen et al. 2017). In this experiment, it was hypothesized that the innate immune training known to be induced by BCG vaccination would enhance the effects of the SIV vaccine. However, BCG-SIV vaccinated animals were shown to be more susceptible to SIV infection, requiring fewer SIV exposures to become infected compared to unvaccinated controls. It was proposed that BCG vaccination caused an expansion of the CD4 + T cell population in addition to trained immunity, giving the virus more opportunities to infect host cells and establish a productive infection.

Besides the studies mentioned above, which either explicitly investigated the role of trained immunity in HIV, or suggested their findings were a result of this phenotype, multiple other HIV studies that were not aimed at exploring trained immunity have coincidentally reported findings analogous to the phenomenon. For example, a study aiming to elucidate the connection between high levels of circulating IL-6 and CVD risk in PLWH found that monocytes isolated from PLWH on ART produced significantly more IL-1β, IL-6, and IL-8 upon ex vivo stimulation with either the proinflammatory lipid species, oxidized low-density lipoprotein (oxLDL) or LPS (Jalbert et al. 2013). In another study, monocytes isolated from untreated PLWH produced significantly more IL-6, IL-1β, CCL3, CCL4, and CCL2 upon ex vivo stimulation with *M. tuberculosis* and monocytes from PLWH on ART produced significantly more IL-1β (Espindola et al. 2018) in agreement with what was shown in ex vivo stimulation of monocytes from PLWH on ART with other secondary stimuli (van der Heijden et al. 2021). As in trained immunity, mechanisms for the observed phenotype were proposed to be rooted in epigenetics, as differential expression of epigenetic enzymes and global epigenetic alterations were observed in the monocytes from PLWH (Espindola et al. 2018). In a similar study, PBMCs from untreated PLWH exhibited increased production of TNFα compared to healthy controls upon ex vivo stimulation with LPS, and PBMCs from uninfected controls that were pre-treated with HIV single-stranded RNA (ssRNA) exhibited similar increased production of TNFα after LPS stimulation (Lester et al. 2008, 2009). While these findings might suggest a trained immunity phenotype, adaptive immune cell activity may have contributed to the response, as whole PBMCs containing T and B cells were utilized in these two experiments. In addition to the trained immunity-like phenotypes observed in monocytes and whole PBMCs, similar phenotypes have also been observed in plasmacytoid dendritic cells and NK cells from PLWH (Abbas et al. 2022; Wang et al. 2020a, b). Other studies have reported enhanced proinflammatory cytokine production in response to secondary stimulation with LPS or other toll-like receptor (TLR) ligands in PBMCs, monocyte-derived macrophages (MDMs), and Kupffer cells exposed to HIV in vitro (Hernandez et al. 2012; Mosoian et al. 2017). In addition, some studies exploring the role of HIV transactivator of transcription (Tat) in modulating cytokine production reported findings functionally similar to those observed in trained immunity. For example, monocytes pretreated with Tat were shown to express IL-6 mRNA to a higher degree than untreated cells after stimulation with LPS (Yim et al. 2009). In a Tat-transgenic mouse model, increased production of IL-1β and increased mRNA expression of IL-1β and TNFα were observed in the ileum of Tat-transgenic mice after in vivo stimulation with LPS in drinking water (Guedia et al. 2016). Together, these findings suggest that HIV itself, HIV ssRNA, or HIV proteins like Tat or gp120 could serve as trained immunity-inducing stimuli (Fig. 1a).

While the previously mentioned studies focused mostly on circulating innate immune cells of the periphery, some studies reporting gene expression signatures of microglia and astrocytes in the context of HIV have coincidentally revealed upregulation of genes associated with trained immunity in the CNS. For example, one study reported upregulation of Lgals3 and Itgax, two genes associated with microglial trained immunity phenotypes (Holtman et al. 2015), in microglia from gp120 transgenic mice (Zheng et al. 2021) and another study reported upregulation of Itgax in human microglia co-cultured with HIV-infected astrocytes (Wang et al. 2008). While it is still unclear how the upregulation of these genes contributes to trained immunity phenotypes in microglia in general, the overlapping expression signature suggests that microglia could display a trained phenotype in the context of HIV infection. In a recent preprint reporting single-cell sequencing signatures of microglia from SIV-infected macaques, multiple microglia clusters from SIV-infected macaques displayed the putative trained microglia signature MHC II^high^/IL-1β^low^/IL-6^low^, suggesting these clusters may represent trained microglia (Godbout et al. 2005; Xu et al. 2024). In addition to microglia, some studies have reported upregulation of genes associated with trained immunity in astrocytes in the context of HIV. To date, only one study has definitively reported the existence of innate immune memory in astrocytes, where a trained immunity-like phenotype was demonstrated in the murine multiple sclerosis model experimental autoimmune encephalomyelitis (EAE) as well as primary human astrocytes treated with training stimuli in vitro (Lee et al. 2024). In this study, it was shown that upregulation of the histone acetyltransferase p300 in astrocytes was crucial for the induction of the trained immunity phenotype, and upregulation of p300 was detected in astrocytes from chronic multiple sclerosis lesions in humans. Interestingly, astrocytes exposed to HIV Tat protein as well as astrocytes infected with HIV in vitro displayed significant upregulation of p300 (Zou et al. 2010). In another study, upregulation of p300 was shown to be associated with HAND progression, albeit in PBMCs (Venkatachari et al. 2017). Although these studies were not aimed at determining whether innate immune memory phenotypes exist in the CNS in the context of HIV, upregulation of these genes may point to the presence of this phenotype in microglia and/or astrocytes (Fig. 1b).

In addition to the previously mentioned gene expression studies, one study found a phenotype more aligned with tolerance in microglia from HIV-Associated Dementia (HAD) patients (Ghorpade et al. 2005). Microglia isolated from these patients produced less TNFα upon LPS stimulation compared to uninfected controls and exhibited lower basal levels of TNFα. These results are likely explained by the fact that the post-mortem brain tissue used in this study was from patients at the end-stage of a chronic inflammatory disease and an argument could be made that this tolerant phenotype may not be observed if analyzing patients who represent the majority of HAND cases today, which are largely ART suppressed and have milder forms of the disease like ANI and MNI (Wang et al. 2020a, b). Aside from this 2005 study which was not focused on innate immune memory, no research has been aimed at determining whether trained or tolerant immunity exists in the context of HIV in the CNS. Any research that has explicitly investigated innate immune memory in HIV has been focused on cells of the periphery. From the little evidence that has been gathered, it seems that this mechanism could be a potential cause or exacerbating factor of the persistent chronic inflammation characteristic of ART-treated HIV. However, since nearly nothing is known about innate immune memory in the HIV-infected CNS, it is unknown whether this phenomenon exists in this system, and if so, whether it is characterized by trained or tolerant immune responses and what the functional implications of these situations would entail.

## Potential drivers of innate immune memory in HIV and HAND

The pathogenesis of HIV in the ART era is largely characterized by aberrant immune activation and chronic systemic inflammation which leaves PLWH at higher risk for a range of comorbidities including cardiovascular, kidney, and hepatic diseases, cancer, and neurocognitive disorders among others (Lerner et al. 2020). Even when HIV replication is successfully suppressed to undetectable levels, circulating biomarkers of inflammation like monocyte chemoattractant protein 1 (MCP-1), neopterin, high sensitivity C-reactive protein (hsCRP), IL-6, D-dimer, TGF-β and others can still be detected at higher levels than in healthy uninfected controls, indicating a persistent inflammatory state (French et al. 2009; Neuhaus et al. 2010; Osuji et al. 2018; Wada et al. 2015). Further, biomarkers of inflammation and coagulation circulating in PLWH on ART like IL-6 and D-dimer are associated with increased morbidity and mortality in these patients (Grund et al. 2016; Hart et al. 2018; Hunt et al. 2016; Kuller et al. 2008). The persistence of inflammatory markers in circulation also coincides with markers of increased innate immune activation in PLWH on ART. For example, markers of monocyte and macrophage activation, soluble CD14 (sCD14) and soluble CD163 (sCD163), are found in circulation at higher levels in PLWH on ART compared to uninfected controls and are associated with mortality (Knudsen et al. 2016; Novelli et al. 2020; Wada et al. 2016). Also, persistent NK cell activation has been reported in PLWH on ART while CD8 + T cell activation was similar to what was observed in healthy uninfected controls (Lichtfuss et al. 2012). In the CNS, aberrant immune activation is apparent in PLWH on ART as measured by increased levels of neopterin, sCD14, IL-6, IL-8, CCL2, CCL3, CXCL10, and IFNγ detected in cerebrospinal fluid (CSF) and these markers are associated with worse cognitive outcomes (Eden et al. 2007; Fleischman et al. 2018; Kamat et al. 2012; Yilmaz et al. 2008). Further, multiple in vivo imaging studies have revealed increased microglial activation in PLWH on ART, even in those who are cognitively asymptomatic, and increased activation levels were correlated with worse cognitive performance in these patients (Garvey et al. 2014; Rubin et al. 2018). These increased markers of immune activation and inflammation in the periphery and CNS are thought to be major drivers of morbidity in HIV, but the underlying causes driving these changes are not completely understood. There are multiple hypotheses that explain the causes of aberrant immune activation and inflammation, which are most likely not mutually exclusive. Interestingly, many of these proposed causes overlap with the stimuli known to drive innate immune memory in the periphery and the CNS, calling into question whether innate immune memory may be involved in, or potentially exacerbating, the chronic inflammation observed in PLWH on ART.

The microbial translocation hypothesis of chronic inflammation in PLWH overlaps with hallmarks of trained immunity. During the first weeks of primary HIV infection, the gut-associated lymphoid tissue (GALT) is one of the most severely affected anatomical sites, experiencing very high levels of CD4 + T cell depletion which is not fully restored by ART (Brenchley et al. 2004; Guadalupe et al. 2003; Marchetti et al. 2008). This leads to a cascade of events that ultimately cause gut epithelial barrier dysfunction and subsequent translocation of proinflammatory microbial products out of the gastrointestinal tract and into circulation, leading to systemic immune activation and inflammation that can persist despite ART (Ancuta et al. 2008; Brenchley et al. 2006; Cassol et al. 2010; Jiang et al. 2009). While it is well understood that these circulating microbial products lead to general systemic immune activation and inflammation, their potential to induce innate immune memory in the context of HIV is poorly understood. β-glucan, which is one of the most well-studied molecules known to drive trained immunity in the periphery and the CNS (Arts et al. 2016; Cheng et al. 2014; Heng et al. 2021; Ifrim et al. 2014; Mitroulis et al. 2018; Moorlag et al. 2020a, b; Quintin et al. 2012; Saeed et al. 2014), can be found in peripheral circulation and in CSF at higher levels in PLWH on ART than uninfected controls and is associated with increased inflammation, immune activation, atherosclerosis, and worse neurocognitive function in these patients (Hoenigl et al. 2015, 2016; Isnard et al. 2021; Mehraj et al. 2020; Morris et al. 2012). This suggests that the increased levels of β-glucan could act as a priming stimulus inducing trained immunity in innate immune cells, exacerbating the systemic inflammation known to be associated with high β-glucan levels. Indeed, the presence of a trained immunity phenotype in monocytes isolated from PLWH on ART was associated with increased levels of circulating β-glucan, supporting this idea (van der Heijden et al. 2021). In addition to its role as a priming stimulus, β-glucan has also been shown to elicit an enhanced proinflammatory cytokine response in monocytes from PLWH compared to controls, suggesting this molecule can both prime cells for trained immunity, and serve as a secondary stimulus for cells that are already trained (Kumar et al. 2023). LPS is also detected in circulation at higher levels than normal in PLWH on ART (Brenchley et al. 2006; d’Ettorre et al. 2011; Merlini et al. 2011). This poses an interesting conundrum when considering innate immune memory, as LPS is known to induce tolerance, calling into question whether LPS-induced tolerance would outweigh training induced by other microbial products like β-glucan. Pertinent to this question, it has been shown that β-glucan stimulation can reverse LPS-induced tolerance through epigenetic reprogramming (Novakovic et al. 2016), suggesting training could be the phenotype that predominates in PLWH on ART with both LPS and β-glucan in circulation. Additionally, LPS is known to induce tolerance or training depending on the concentration of the stimulus (Morris and Li 2012), so at low concentrations, LPS could induce training in the context of chronic HIV. While the current evidence seems to point toward training as the predominant phenotype of innate immune memory in HIV, more studies would be needed to conclusively determine this, especially considering the variety of inflammatory mediators that could be circulating in PLWH simultaneously.

Another hypothesis explaining chronic inflammation in PLWH implicates the low-level production and secretion of viral proteins from the persistent HIV reservoir as inflammatory mediators. Despite the ability of ART to inhibit viral replication and reduce viremia, transcription and translation from integrated viral genomes still occur spontaneously, causing viral proteins including gp120, Nef, Tat, Vpr, and others to be produced and secreted from infected cells (Ferdin et al. 2018; Hoshino et al. 2007; Lee et al. 2016; Matsunaga et al. 2019; Mediouni et al. 2012; Rychert et al. 2010). Interestingly, viral proteins can also be detected in CSF and brain tissue from PLWH on ART, indicating the potential for direct interactions with CNS resident cells like microglia and astrocytes (Donoso et al. 2022; Henderson et al. 2019; Johnson et al. 2013). Each of these proteins has been shown to induce an inflammatory response in bystander cells characterized by the production of IL-1β, IL-6, TNFα and other proinflammatory factors (Ben Haij et al. 2015; Cheung et al. 2008; Del Corno et al. 2014; Hoshino et al. 2010; Mukhamedova et al. 2019; Olivetta et al. 2003; Pu et al. 2003; Roux et al. 2000). Since these proteins are known to cause an inflammatory response, it is reasonable to assume they could also induce innate immune memory. In fact, some strong evidence mentioned previously explicitly implicates Nef as an inducer of trained immunity in myeloid cells (Dubrovsky et al. 2022). In addition, Tat has been shown to induce trained immunity-like phenotypes in primary human monocytes treated with Tat ex vivo and in Tat-transgenic mice (Guedia et al. 2016; Yim et al. 2009). Although it was used as a secondary stimulus, gp120 was shown to induce increased neuroinflammation in aged mice, implicating it as a potential exacerbating factor in aging PLWH (Abraham et al. 2008). While Vpr and gp120 have not been shown to induce trained immunity directly, the upregulation of IL-1β they (Cheung et al. 2008; Guha et al. 2012; Mamik et al. 2017) and other viral proteins cause could be a potential priming stimulus for trained immunity alone as IL-1β itself has been shown to induce trained immunity in human monocytes in vitro (Arts et al. 2018; Moorlag et al. 2020a, b). This suggests a variety of viral proteins released from infected cells could either induce training directly through interactions with bystander cells, or through an indirect mechanism via induction of IL-1β signaling. Additionally, some evidence points to the potential of HIV infection itself inducing a training-like state in liver-resident macrophages (Mosoian et al. 2017; Zhang et al. 2019), but it is still unclear whether other innate immune cells infected with HIV would exhibit a trained or tolerant response programmed by infection itself.

Lipid abnormalities characterized by altered levels of several cholesterol and lipoprotein species are another potential driver of chronic inflammation in PLWH on ART and have been linked to the increased incidence of atherosclerotic CVD in this population (Dirajlal-Fargo and Funderburg 2022; Funderburg and Mehta 2016). One common observation of the abnormal lipid profiles detected in PLWH on ART includes increased levels of the lipid species, oxLDL (Duong et al. 2006; Kelesidis et al. 2016; Zidar et al. 2015). In general, oxLDL is known to be proinflammatory, and is associated with atherosclerotic CVD in the general HIV-uninfected population (Gao et al. 2017; Trpkovic et al. 2015). Increased levels of oxLDL have also been associated with markers of immune activation and inflammation in PLWH on ART, suggesting oxLDL is a potential driver of chronic inflammation in this state (Kelesidis et al. 2016; Zidar et al. 2015). Interestingly, oxLDL has also been shown to induce trained immunity in human monocytes (Bekkering et al. 2014, 2018; Christ et al. 2018; Keating et al. 2020a, b), indicating yet another factor associated with chronic inflammation in PLWH that could contribute to a trained immunity phenotype (Fig. 1a). In a study that reported results functionally similar to trained immunity in monocytes from PLWH on ART, secondary stimulation with oxLDL ex vivo induced an increased proinflammatory response compared to monocytes from uninfected controls (Jalbert et al. 2013). Since the general inflammatory conditions associated with HIV in vivo were technically the primary stimulus for training in this case, oxLDL was only analyzed as a secondary stimulus which suggests that in an already trained state, oxLDL may contribute to the chronic systemic inflammation observed in PLWH on ART. However, it is reasonable to assume oxLDL could act as a primary stimulus to train cells in the context of HIV as it has been shown multiple times to induce training when used as a primary stimulus (Bekkering et al. 2014, 2018; Christ et al. 2018; Keating et al. 2020a, b). With this evidence, it is reasonable to assume that this inflammatory lipid species known to be present at high levels in PLWH on ART could be yet another factor driving trained immunity along with secreted viral proteins and microbial translocation products.

From the observations mentioned above, it is clear that many factors circulating in PLWH on ART could lead to a trained immunity phenotype in cells of the periphery like monocytes and macrophages. While many of these factors are known to induce trained immunity in the periphery, this does not rule out the possibility that they could induce trained immunity in the CNS. For one, some of the potential priming stimuli that are detected in the periphery are also presumably present in the CNS as several are detected in the CSF of PLWH on ART (Donoso et al. 2022; Henderson et al. 2019; Hoenigl et al. 2016; Johnson et al. 2013), making it likely that similar mechanisms of priming could occur there. While some of these stimuli, like viral proteins, may be produced by cells in the CNS from the persistent viral reservoir housed there, others may enter through the BBB, which is known to exhibit disrupted integrity in PLWH, albeit to a lower level in ART suppressed patients (Anesten et al. 2021; Calcagno et al. 2017). It is also well known that stimulation of the innate immune system in the periphery can cause microglial activation and neuroinflammation in the CNS, reviewed in detail here: (Hoogland et al. 2015; Sun et al. 2022). This is evident when considering that many of the experimental demonstrations of trained immunity in microglia are induced by applying priming stimuli to the periphery and not directly into the CNS (Chen et al. 2008; Cunningham et al. 2005; Godbout et al. 2005; Heng et al. 2021; Henry et al. 2009; Wendeln et al. 2018). Therefore, a trained response in the periphery, while exacerbating chronic inflammation systemically, could spill over into the CNS, contributing to the neuroinflammation associated with HAND by priming microglia and/or astrocytes for trained immunity (Fig. 1b). Through these mechanisms, it is possible that trained immunity could occur in both the periphery and the CNS of PLWH on ART contributing to or exacerbating the chronic inflammation characteristic of the disease.

## Clinical implications of innate immune memory in HIV and HAND

In general, trained immunity and tolerance can be either protective or maladaptive depending on the context of the disease state (Lajqi et al. 2023). For trained immunity specifically, clinical implications vary depending on the chronicity of the condition in which the inflammatory response is taking place. For example, the trained response is largely seen as protective in the context of an acute infection as trained innate immune cells are programmed to clear the infection more efficiently. This is evident in humans who were vaccinated with the known training stimulus, BCG, then infected with a live attenuated yellow fever virus (YFV) vaccine strain one month later (Arts et al. 2018). BCG-vaccinated individuals displaying trained immunity cleared the YFV infection more efficiently leading to significantly lower viremia, providing an example of the way trained immunity can be protective in acute infections. In addition to this example, experiments in animal models support this idea, as mice pre-treated with a training stimulus are able to clear acute infections more efficiently than naïve mice in an adaptive immune system-independent manner, leading to greater survival (Bistoni et al. 1988; Kleinnijenhuis et al. 2012; Munoz et al. 2010; Quintin et al. 2012). However, in the context of a chronic disease state, trained immunity is thought to be maladaptive. One of the most widely recognized examples of this is in atherosclerosis, where the increased proinflammatory signaling characteristic of trained immunity is thought to contribute to the chronic inflammatory nature of the disease and facilitate cardiovascular disease progression (Flores-Gomez et al. 2021; Zhong et al. 2020). The stimulation of monocytes, macrophages, and endothelial cells by endogenous atherogenic molecules like oxLDL and lipoprotein (a) is known to induce trained immunity (Bekkering et al. 2014; van der Valk et al. 2016) and the chronic nature of this stimulation in the context of atherosclerosis is thought to keep these cells in a trained state leading to the non-resolving vascular inflammation characteristic of the condition. In the CNS, evidence has also shown that in a chronic neurodegenerative environment like Alzheimer’s, trained immunity is maladaptive as it exacerbates markers of disease progression (Wendeln et al. 2018). In the case of tolerance, both protective and maladaptive effects can be seen simultaneously, and this is well exemplified during sepsis. On one hand, innate immune tolerance during sepsis can prevent a lethal hyperinflammatory response to the ongoing systemic infection, protecting the individual from excessive tissue damage (Lopez-Collazo and del Fresno 2013). On the other hand, this phenotype can leave the individual vulnerable to secondary infections, which often occur during sepsis (van Vught et al. 2016). While the roles of training and tolerance have been considered in multiple disease states, the clinical implications of these phenotypes are not well understood in the context of HIV.

Considering the direct evidence for trained immunity in the context of HIV infection (Dubrovsky et al. 2022; Faia et al. 2021; van der Heijden et al. 2021; Vittori et al. 2024) and the observations of analogous phenotypes suggesting its possibility (Espindola et al. 2018; Guedia et al. 2016; Hernandez et al. 2012; Jalbert et al. 2013; Lester et al. 2008, 2009; Mosoian et al. 2017; Yim et al. 2009), the question remains; what would be the clinical implications of trained immunity in chronic HIV infection? It is tempting to propose trained immunity as an explanation for the persistent chronic inflammation that occurs in ART-suppressed PLWH. While it is unlikely that trained immunity is the sole factor to blame in this setting, it is possible that it contributes to and exacerbates the persistent inflammation observed. In this scenario, a multitude of inflammatory factors circulating in PLWH on ART could induce trained immunity both in the periphery and the CNS and the continued circulation of these factors could lead to subsequent non-resolving inflammation and immune activation exacerbated by training. In this sense, a model similar to what has been described for trained immunity in atherosclerosis could exist (Flores-Gomez et al. 2021) where training is caused by constant exposure to circulating proinflammatory factors which then causes innate immune cells to continuously overreact to these same factors (Fig. 1c). In HIV, this could be caused by constant exposure to viral proteins, microbial translocation products, or inflammatory lipid species as mentioned previously.

A more concerning possibility proposed by Dubrovsky et al. speculates that even if a cure for HIV is eventually developed, long-lasting trained immunity could leave cured individuals in a persistent inflammatory state (Dubrovsky et al. 2022). In this scenario, the benefits of a sterilizing or functional HIV cure may not be fully realized as comorbidities like CVD and HAND could still exist due to a continued state of persistent inflammation driven by trained immunity (Fig. 1c). However, important questions remain that would determine whether this scenario could realistically exist, primarily; what is the true durability of this phenotype in the absence of priming stimuli? Specifically, if an HIV cure ameliorated the circulation of viral proteins, microbial translocation products, and proinflammatory lipid species, would innate immune memory dissipate? In the periphery, the trained immune response has been shown to last at least 1 year after BCG vaccination, however, further timepoints were not tested in this study, so it is possible that the phenotype remains durable even longer (Kleinnijenhuis et al. 2014). In the CNS, a primed phenotype was apparent 6 months after a single training stimulus, but again, further timepoints were not tested so the true upper limit of durability is still unclear (Wendeln et al. 2018). Regardless, the ability for trained immunity to be programmed at the bone marrow progenitor level, as opposed to just in short-lived monocytes and mature macrophages, theoretically allows for long-term memory in the periphery (Cirovic et al. 2020; Kaufmann et al. 2018). In the CNS, microglia are quite long-lived as one study proposed that the average microglial cell is 4.2 years old and that some can live more than 20 years (Reu et al. 2017), allowing for the possibility of long-term epigenetic memory storage. However, the epigenetic mechanism of innate immune memory is inherently reversible, as opposed to adaptive immune memory which is encoded in the genome sequence. Because of this, one might assume that this epigenetic state would be quickly reversed after priming stimuli are removed, which could be true, although the epigenetic reprogramming of immune cells in other disease states has been shown to persist after cure or resolution of disease. For example, in the context of hepatitis C virus (HCV), another chronic viral infection, it has been observed that clinical cure with direct-acting antivirals (DAA) does not normalize the altered proinflammatory state caused by chronic viral infection (Aregay et al. 2021) and even when patients are cured during the acute phase of infection, levels of soluble inflammatory mediators do not completely normalize (Khera et al. 2022). In line with these observations, epigenetic reprogramming of immune cells that occurs during HCV is not fully reversed after cure, providing a potential explanation for the persistence of inflammation (Perez et al. 2019; Yates et al. 2021). Described as “epigenetic scarring”, this phenomenon has been proposed as a potential driver of the persistent risk of hepatocellular carcinoma (HCC) after HCV cure. In addition to HCV, recent studies have revealed trained immunity phenotypes in myeloid cells of patients recovering from COVID-19 as well as long-term reprogramming of hematopoietic stem cells during severe COVID-19 which persisted for at least 1 year after recovery, potentially contributing to the pathogenesis of “long COVID” (Cheong et al. 2023; You et al. 2021). While these examples are obviously not equivalent to chronic HIV infection, they provide proof-of-principle that even after clinical cure or recovery from acute infection, and therefore removal of the stimuli which caused an epigenetically programmed immune state, the phenotype is not necessarily reversed and continued morbidity can persist. It has also been observed in animal models that epigenetic-driven immune states like trained immunity persist even when the primary training stimulus is absent. This is evident in the fact that transplant of bone marrow from mice trained in vivo with HIV Nef EVs into naïve mice results in durability of the trained phenotype for at least 11 weeks in those naïve mice (Dubrovsky et al. 2022). Thus, when trained cells are transferred to an animal that is not encountering the primary stimulus, training remains. Again, while this does not exactly recapitulate an HIV cure, it demonstrates that after removing the primary stimulus, or in the case of HIV, removing the factor causing the circulation of primary stimuli, epigenetically programmed trained immunity could persist, presenting a potentially serious challenge that should be considered in HIV cure development.

Even if trained immunity can dissipate after HIV cure, the phenotype may be more likely to remain durable in older adults living with HIV (OALWH), especially in the CNS, leading to the persistence of conditions like HAND and other inflammation-associated comorbidities after cure. With the widespread use of ART, the average lifespan of PLWH has increased, causing an increase in the number of OALWH compared to the pre-ART era (Govender et al. 2021). Indeed, the majority of PLWH in the US are over 50 years old (CDC 2023), and globally, the number of PLWH over 50 years old tripled between 2000 and 2016 and this increase is expected to continue growing as PLWH on ART are living longer than ever before (Autenrieth et al. 2018). In normal aging, a state of low-grade chronic inflammation, which has been termed ‘inflammaging’, develops and is thought to contribute to a range of age-related diseases (Franceschi and Campisi 2014). The underlying drivers of chronic inflammation in aging are not fully understood, but one proposed cause is a constant stimulation of the innate immune system with sterile inflammatory mediators (Franceschi et al. 2017). From this description, it is tempting to assume that if the innate immune system is already in a trained state from a chronic infection like HIV, inflammaging might be exacerbated. In OALWH, curing HIV and thus removing the root cause of the stimuli which induced trained immunity in the first place may not reverse the phenotype since there is an ongoing state of innate immune activation (inflammaging) superimposed onto the already existing state of trained immunity. This could be especially damaging in the CNS, where normal aging alone is already known to prime microglia for trained immunity or training-like phenotypes (Abraham et al. 2008; Godbout et al. 2005; Henry et al. 2009; Huang et al. 2008; Wynne et al. 2010). In the case of OALWH, the CNS could house microglia and/or astrocytes trained by both chronic HIV infection and aging, which could lead to a larger population of trained CNS cells and thus a higher burden of neuroinflammation. After cure, the CNS cells trained by chronic HIV infection might remain in the trained state due to the constant stimulation with inflammatory mediators present in normal aging, potentially leading to the persistence and even progression of HAND. Since much of this is highly speculative at this point, more research is needed to determine the full implications of innate immune memory in the periphery and the CNS in the context of chronic HIV, especially in OALWH. If evidence suggests that trained immunity could persist after an HIV cure, this should be considered in the development of cure strategies, as it may be necessary to reverse trained immunity either during or after the cure regimen to realize the full benefits of the cure.

## Concluding remarks

Because so little is known about innate immune memory in the context of HIV, the potential clinical implications are left largely to speculation. However, the limited evidence available suggests that trained immunity does exist in this context, warranting a more thorough investigation into the many outstanding questions surrounding this immune state and its contribution to chronic HIV pathogenesis in the ART era. It is possible that a combination of inflammatory mediators known to be present in PLWH on ART like microbial translocation products, viral proteins, and inflammatory lipid species could cause trained immunity phenotypes in both the periphery and the CNS. Indeed, many of the proposed causes of chronic inflammation in PLWH on ART overlap with what has already been shown to induce trained immunity like B-glucan, oxLDL, and HIV Nef. The existence of trained immunity in ART-treated HIV could contribute to or exacerbate the chronic inflammation and innate immune activation associated with the condition, and the potential for this long-lasting memory to persist after curing HIV could present more challenges in the future. For these reasons, future studies should focus on determining the stimuli that cause innate immune memory phenotypes in the context of HIV and elucidating the mechanisms by which this occurs to understand if and how this phenotype contributes to HIV comorbidities like CVD and HAND. Understanding these mechanisms may open the door for new therapeutic strategies aimed at reversing the trained immunity phenotype to potentially prevent chronic inflammation and immune activation with the goal of alleviating the burden of HIV comorbidities.

## Data Availability

No datasets were generated or analysed during the current study.
